# Biparametric MRI with deep learning for the preoperative prediction of muscle invasion in patients with bladder cancer: a multicenter study

**DOI:** 10.1186/s13244-026-02430-1

**Published:** 2026-11-09

**Authors:** Ziyi Liu, Yilan Hu, Jiegeng Lyu, Lei Deng, Shuiqing Yang, Xiang Huang, Xiaohui Duan, Weijing Zhang, Bingsheng Huang, Jianpeng Li, Yujian Zou

**Affiliations:** 1 Department of Radiology, The Tenth Affiliated Hospital of Southern Medical University (Dongguan People’s Hospital), Dongguan, China; 2 Department of Radiology, Daojiao Hospital of Dongguan, Dongguan, China; 3 Medical AI Lab, School of Biomedical Engineering, Shenzhen University Medical School, Shenzhen University, Shenzhen, China; 4 Department of Radiology, Sun Yat-Sen Memorial Hospital, Sun Yat-Sen University, Guangzhou, China; 5 Imaging Department, Sun Yat-Sen University Cancer Center, State Key Laboratory of Oncology in South China, Collaborative Innovation Center for Cancer Medicine, Guangzhou, China

**Keywords:** Deep learning, Magnetic resonance imaging, Multicenter studies as topic, Neoplasm invasiveness, Urinary bladder neoplasms

## Abstract

**Objectives:**

This study aimed to develop and validate a deep learning model that integrates T2-weighted imaging (T2WI) and diffusion-weighted imaging (DWI) for the noninvasive prediction of muscle invasion in bladder cancer.

**Materials and methods:**

A total of 308 bladder tumors from 254 patients were retrospectively collected from three institutions. Of these, 177 tumors from Center 1 were partitioned via stratified sampling into a training set (*n* = 128) and an internal validation set (*n* = 49). The remaining two centers contributed 59 and 72 tumors, respectively, which served as external validation cohorts. A dual-branch 3D deep convolutional neural network (Joint model) based on biparametric MRI (bp-MRI) was developed using the training data and compared with a deep learning model based on T2WI (T2WI model) and VI-RADS assessments by radiologists. Diagnostic performance was evaluated using the area under the receiver operating characteristic curve (AUC), accuracy, sensitivity, and specificity.

**Results:**

The Joint model achieved mean AUCs of 0.979 ± 0.007 in the internal validation cohort, and 0.907 ± 0.013 and 0.916 ± 0.021 in the external validation cohorts. It outperformed the T2WI model in all cohorts and achieved higher AUCs than radiologists across all validation cohorts. Notably, for equivocal VI-RADS 2–3 lesions, the Joint model yielded higher accuracy than the T2WI model in external validation.

**Conclusion:**

Compared with the T2WI model and VI-RADS assessment, the Joint model demonstrated better diagnostic performance and generalizability for predicting muscle invasion in patients with bladder cancer.

**Key Points:**

***Question*** Reliable noninvasive tools for preoperative prediction of muscle invasion in bladder cancer are lacking, as TURBT-based staging carries procedural risks and frequent understaging.

***Findings*** A biparametric MRI deep learning model achieved AUCs of 0.907–0.979 across internal and external validation cohorts, outperforming T2WI-only models and VI-RADS.

***Critical relevance statement*** Contrast-free biparametric MRI combined with deep learning enables accurate, generalizable preoperative prediction of muscle invasion, offering particular value for equivocal VI-RADS 2–3 lesions and supporting noninvasive clinical decision-making.

**Graphical Abstract:**

**Figure:**
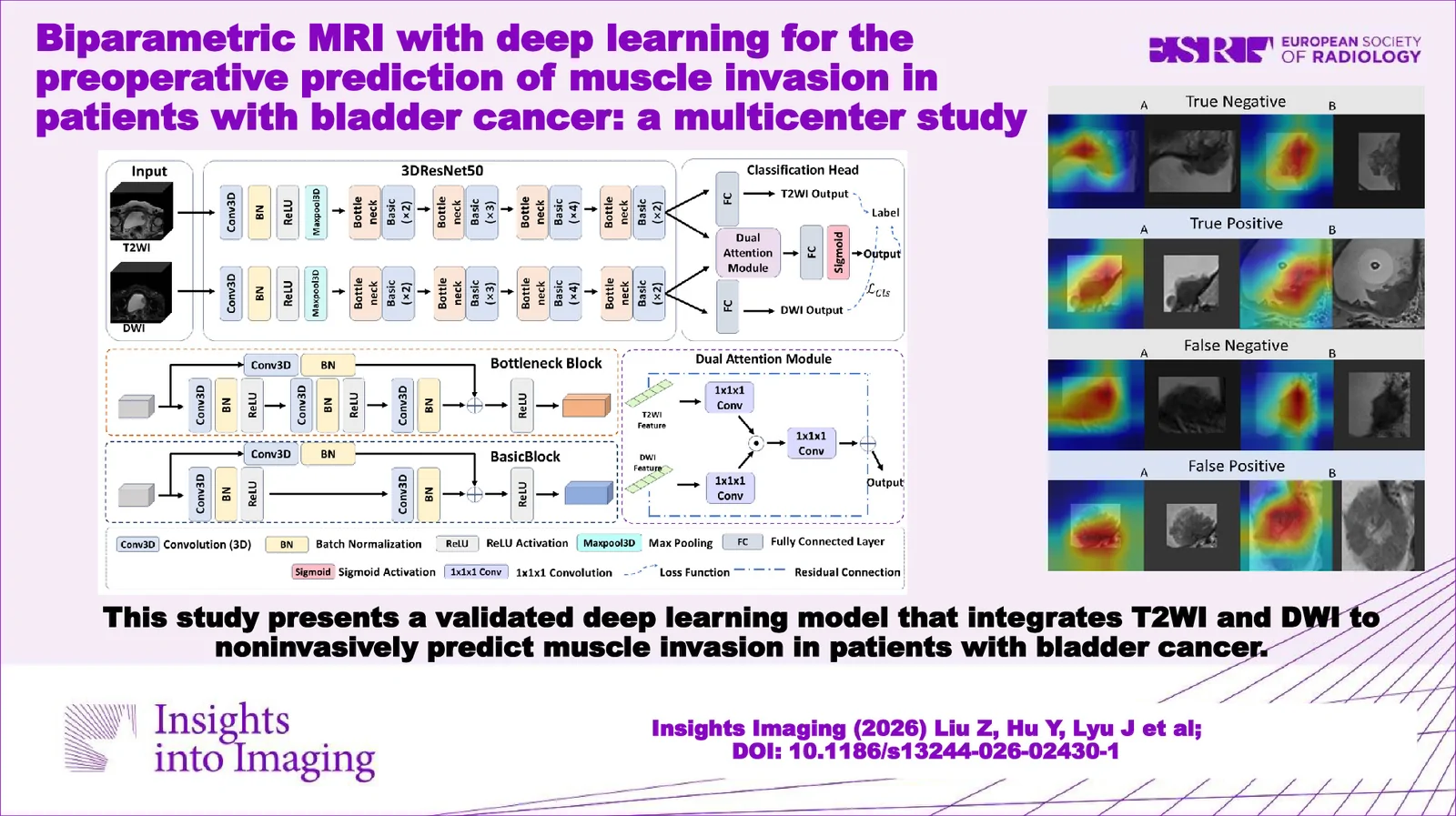

## Introduction

Muscle invasion status serves as a key determinant for treatment planning and prognosis in bladder cancer [1, 2]. Currently, staging relies primarily on transurethral resection of bladder tumor (TURBT) and cystoscopic evaluation. Cystoscopy is a routine outpatient diagnostic tool; however, it is operator-dependent, exhibits low sensitivity in detecting small papillary and flat lesions, and performs poorly in defining resection margins [3]. Moreover, studies have shown that up to 25% of TURBT-based assessments understage disease, misclassifying MIBC as NMIBC [4]. Additionally, TURBT carries procedural risks, including bleeding, infection, and bladder perforation [5]. This points to a clinical challenge: achieving reliable preoperative prediction of muscle invasion through noninvasive imaging to reduce dependence on invasive staging procedures.

Multiparametric MRI (mp-MRI) and the Vesical Imaging-Reporting and Data System (VI-RADS) have emerged as promising noninvasive alternatives for local staging [6, 7]. Within this framework, T2WI provides anatomical detail, DWI offers functional characterization of tissue cellularity, and DCE supplies perfusion information [8]; however, the definitive distinction between non-muscle-invasive bladder cancer (NMIBC) and muscle-invasive bladder cancer (MIBC) within VI-RADS typically requires DCE integration [8]. The mandatory use of gadolinium-based contrast agents introduces well-documented risks of nephrogenic systemic fibrosis, which limits applicability in patients with renal impairment [9]. Biparametric MRI (bp-MRI), combining T2WI and DWI without contrast administration, has demonstrated comparable diagnostic performance to mp-MRI in recent investigations [10–12], suggesting a contrast-free option. Nevertheless, existing deep learning approaches predominantly utilize T2WI alone [13–15], thereby neglecting the complementary diffusion information that DWI provides, and these single-sequence models exhibit marked performance degradation in external validation cohorts. Although a multiparametric deep learning model has shown superior accuracy [16], it depends on full three-sequence input including DCE. Consequently, an unresolved question remains: whether a deep learning model built exclusively on bp-MRI can achieve consistent cross-institutional generalization, particularly for equivocal VI-RADS 2–3 lesions that are prone to interobserver inconsistency [17–19].

In this study, we developed and validated a dual-branch 3D deep convolutional neural network (Joint model) that integrates T2WI and DWI, and compared its diagnostic performance against radiologists’ VI-RADS assessments and a single-sequence T2WI model across internal and external validation cohorts. We hypothesized that the Joint model would achieve diagnostic performance comparable to or better than that of VI-RADS assessment and the T2WI-based DL model. Specifically, we expected the model to demonstrate higher diagnostic accuracy in cases scored as VI-RADS 2 and 3, along with improved generalizability in external validation cohorts.

## Materials and methods

### Patients

This retrospective multicenter study was approved by the institutional review boards of all participating institutions, with a waiver of informed consent. A total of 308 bladder tumors from 254 patients were included from three institutions: Dongguan People’s Hospital, Sun Yat-sen University Cancer Center, and Sun Yat-sen Memorial Hospital. Each tumor was treated as an independent sample for subsequent modeling and analysis in this study. All patients underwent preoperative mp-MRI, including T2-weighted imaging (T2WI), diffusion-weighted imaging (DWI), and dynamic contrast-enhanced imaging (DCE).

Regarding the reference standard, TURBT is the primary method for diagnosing and staging bladder cancer when initial tests suggest bladder tumors [20]. For patients with multiple tumors who underwent TURBT, all lesions were labeled as NMIBC if the pathological results of all lesions confirmed non-muscle-invasive bladder cancer; the case was excluded if any lesion was pathologically confirmed as muscle-invasive bladder cancer (MIBC).

**Fig. 1:**
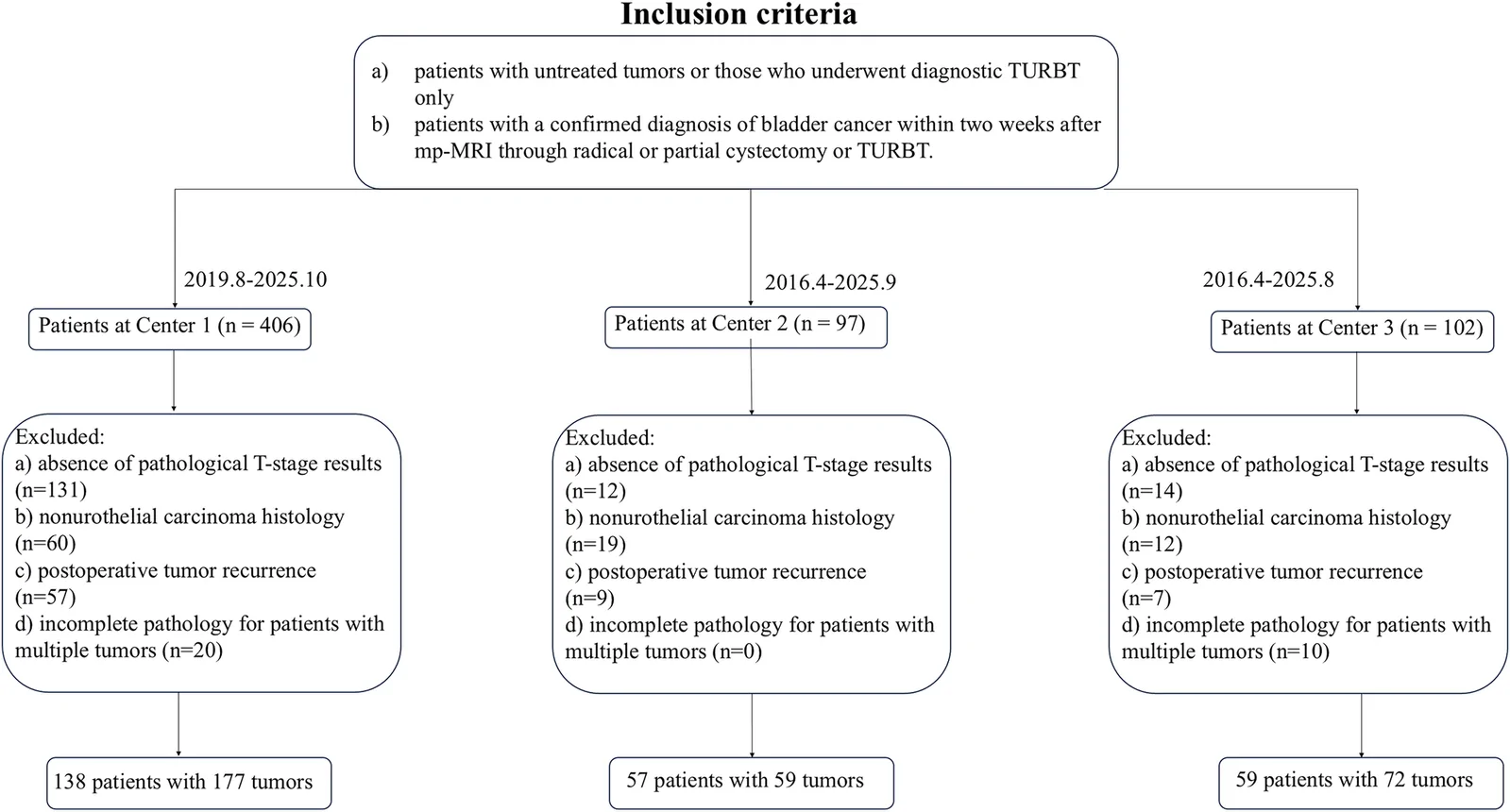
Flowchart of patient enrollment in the study

The data from Center 1 were divided via stratified sampling at a 7:3 ratio into training and internal validation cohorts. Additionally, two independent external validation cohorts were established for model evaluation. The inclusion criteria were as follows: (1) patients with untreated tumors or those who underwent diagnostic TURBT only, and (2) patients with a confirmed diagnosis of bladder cancer within 2 weeks after mp-MRI through radical or partial cystectomy or TURBT. The exclusion criteria were as follows: (1) absence of pathological T-stage results, (2) nonurothelial carcinoma histology, (3) postoperative tumor recurrence, and (4) incomplete pathology for patients with multiple tumors. The data screening process is illustrated in Fig. 1.

### VI-RADS score evaluation

In all three centers, VI-RADS scoring followed the routine clinical workflow: an initial interpretation was performed by a junior radiologist with 5 years of experience in genitourinary imaging, followed by confirmation by a senior radiologist. To simulate this clinical process, two independent reader groups were formed for this study, each consisting of one junior radiologist and one senior radiologist. Both groups (Reader 1 and Reader 2) assessed bladder tumors in the validation cohorts using T2WI, DWI, and DCE. All readers were blinded to the pathological results and study design to ensure independent evaluation.

### Development and validation of the DL model

#### Image preprocessing

A radiologist manually annotated the bladder tumors using ITK-SNAP (version 3.4.0; www.itksnap.org) [21]. Rectangular bounding boxes were drawn on the start and end slices of the lesion in the axial plane to define its z-axis span. The slice with the most prominent tumor appearance was used to define the x and y dimensions of a 3D box enclosing the entire tumor, which served as the basis for cropping. To evaluate annotation stability, a randomly selected subset of samples was re-annotated by the same radiologist after a washout period of 6 months to assess intra-observer agreement. Additionally, a second radiologist independently delineated the same subset to assess interobserver agreement. All volumes of interest (VOIs) were carefully verified to ensure complete tumor coverage. The preprocessing steps, including cropping, normalization, and augmentation, are detailed in the Supplementary Material.

### Development of deep learning models

We constructed a dual-sequence fusion model (Joint model) and three single-sequence T2WI-based models for comparison: a 3D ResNet-50 (T2WI model) [22], a 3D ResNet-18, and a 3D DenseNet-121 [23]. All models employed ReLU activation functions and a softmax output layer to produce prediction probabilities.

**Fig. 2:**
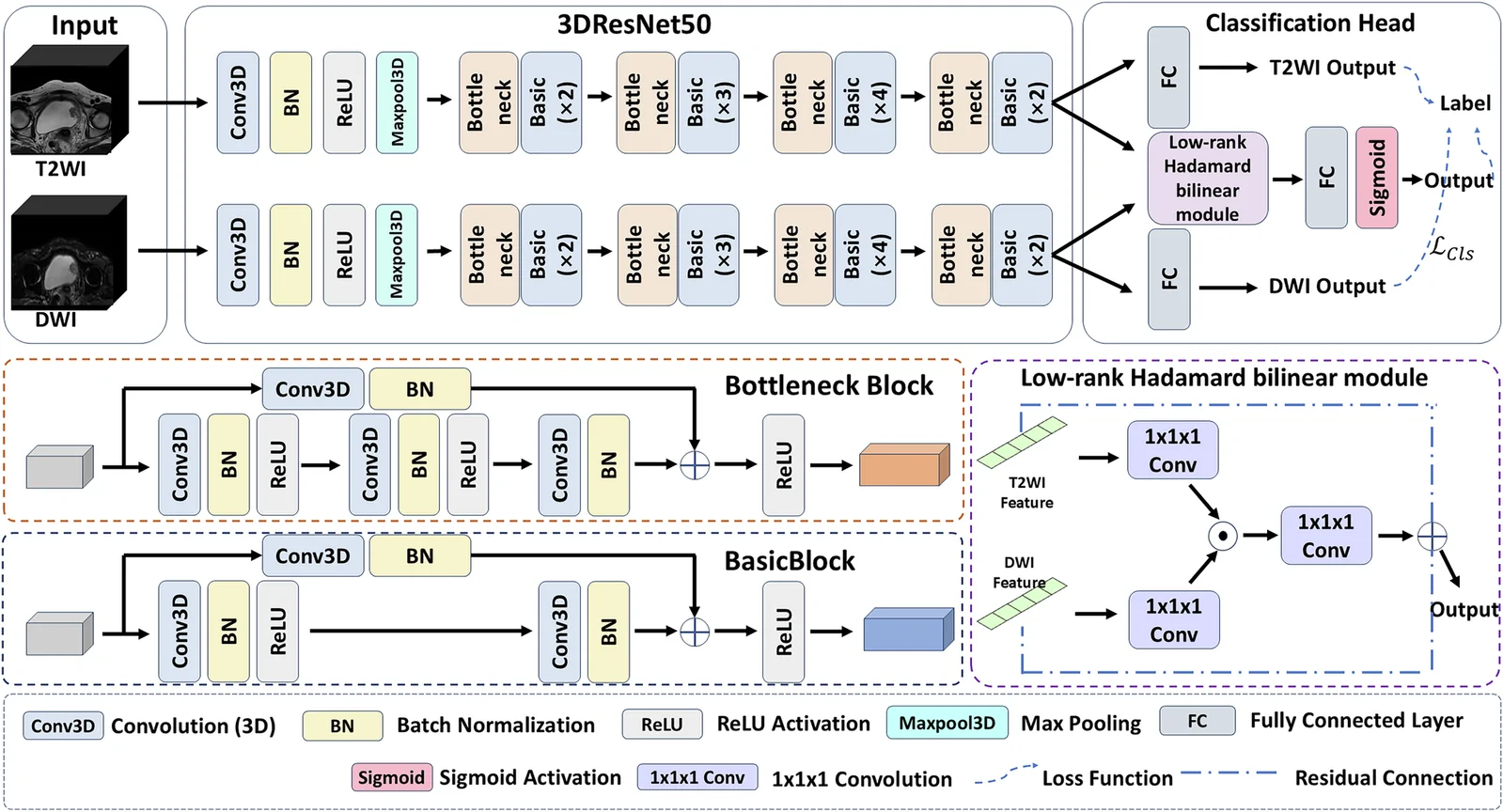
Architecture of the Joint model. T2WI and DWI volumes are processed through independent 3D ResNet-50 encoders. Each branch includes an auxiliary classification head (deep supervision). Encoder feature maps are fused via a low-rank Hadamard bilinear module (1 × 1 × 1 convolutions for low-rank projection, element-wise multiplication, and residual connection) and passed to a fully connected layer for the final prediction. Conv, 3D convolution; BN, batch normalization; ReLU, rectified linear unit; FC, fully connected layer; Sigmoid, sigmoid activation

For the Joint model, T2WI and DWI sequences were input into separate 3D ResNet-50 encoders to extract high-dimensional feature maps. Each branch included an auxiliary classification head comprising a fully connected layer to generate independent T2WI and DWI predictions, enforcing deep supervision. The encoder feature maps from both branches were additionally fed into a low-rank Hadamard bilinear fusion module. Within this module, both sequences were first projected into a shared low-rank subspace via 1 × 1 × 1 convolutions. The projected features were then multiplied element-wise to capture second-order inter-sequence interactions, and the result was projected back to the original channel dimension. A residual connection added the original single-sequence features to this fused representation, preserving single-sequence information. By the Eckart–Young–Mirsky theorem, this low-rank factorization approximates the full bilinear interaction matrix while preserving pairwise inter-sequence feature interactions [24, 25]. The fused representation was subsequently passed through a fully connected layer with sigmoid activation to generate the final prediction. Classification losses were applied to the fused output and both auxiliary outputs to jointly optimize the entire network. The model architecture is illustrated in Fig. 2.

### Model training and validation

The T2WI model and the Joint model were both trained on the training cohort from Center 1 and validated on the three independent validation cohorts from Centers 1, 2, and 3. To address the class imbalance between the MIBC and NMIBC cases, an enhanced version of Focal Loss—Equalized Focal Loss (EFL) [26, 27]—was employed as the loss function. Unlike conventional Focal Loss, which down-weights easy samples and focuses on hard-to-classify samples globally, EFL dynamically adjusts the loss weights for each class based on the degree of intrinsic imbalance, thereby improving learning performance across imbalanced categories.

For the Joint model, a deep supervision strategy was additionally implemented to ensure that each encoder branch learned discriminative unimodal representations before fusion. Small fully connected heads were attached to both the T2WI and DWI branches, producing auxiliary predictions alongside the main fused output. This multitask formulation prevents the fusion layer from relying on a single dominant modality and encourages complementary feature learning across sequences.

The Adam optimizer was used to minimize the EFL loss, with a cosine annealing learning rate schedule oscillating between 1e-4 and 1e-6 over 120 training epochs. The model outputs the probability of muscle-invasive bladder cancer (MIBC).

To assess the robustness of initialization, each architecture was independently trained ten times from scratch using different random seeds. The mean ± standard deviation (SD) of performance metrics across the ten runs is reported.

All experiments were conducted using Python 3.6.15 on a workstation running Ubuntu 20.04. The hardware configuration included an Intel Xeon E5–2650 V3 CPU (2.30 GHz, 20 cores) and an NVIDIA GeForce GTX 1080 Ti GPU.

### Feature visualization

To visualize model decision-making, we applied gradient-weighted class activation mapping (Grad-CAM) [28], which illustrates the CNN’s spatial attention by generating heatmaps based on gradients of the final convolutional layer.

For feature analysis, we employed the t-distributed stochastic neighbor embedding (t-SNE) algorithm [29]. As a data-driven dimensionality reduction method, t-SNE effectively projects high-dimensional feature structures into low-dimensional space. In this study, t-SNE reduced the 2048 features extracted by the DL model to two dimensions, which were then visualized in 2D space to demonstrate feature distribution differences between NMIBC and MIBC patients.

### Statistical analysis

Statistical analyses were performed using MedCalc for Windows, version 20.009 (MedCalc Software). Categorical variables were compared using the chi-square test, and continuous variables were compared using independent sample *t*-tests. Interobserver agreement on VI-RADS scores was evaluated using Cohen’s kappa coefficient. The diagnostic performance of the deep learning models and VI-RADS was assessed using the area under the receiver operating characteristic curve (AUC) with 95% confidence intervals (CIs). The optimal cutoff values were determined by maximizing the Youden index, and metrics such as accuracy (ACC), sensitivity (SEN), and specificity (SPE) were calculated.

For comparisons among deep learning models, paired *t*-tests were performed on the ten AUC values obtained from the ten independent training runs. For comparisons between deep learning models and radiologists, a one-sample *t*-test was used to compare the AUC values of each model against the AUC value of Reader 1 or Reader 2, respectively. For the subset analysis of tumors scoring VI-RADS 2 and 3, one model instance was randomly selected from the ten trained runs for each architecture; AUC comparisons were performed using DeLong’s test, and accuracy comparisons were performed using McNemar’s test. A two-tailed *p*-value of < 0.05 was considered statistically significant.

## Results

### Patient characteristics

This study included 308 bladder tumors from 254 patients, with each tumor treated as an independent unit of analysis. Specifically, the data were distributed as follows: Dongguan People’s Hospital—138 patients (177 tumors, MIBC:NMIBC = 33:144), Sun Yat-sen University Cancer Center—57 patients (59 tumors, MIBC:NMIBC = 33:26), and Sun Yat-sen Memorial Hospital—59 patients (72 tumors, MIBC:NMIBC = 22:55). Detailed clinical information is provided in Tables 1 and E2. As shown in Table 1, there were no significant differences in age or sex among the training and internal validation cohorts (Center 1) and the external validation cohorts (Centers 2 and 3).

**Table 1:** Patient data characteristics (tumor-based summary)

Characteristics	Number of tumors						
	Training cohort (Center 1) (*n* = 128)	Internal validation cohort (Center 1) (*n* = 49)	*p*-value	External validation cohort (Center 2) (*n* = 59)	*p*-value	External validation cohort (Center 3) (*n* = 72)	*p*-value
Age (years)	65.66 ± 10.66	66.88 ± 11.87	0.513	64.15 ± 11.94	0.387	64.00 ± 9.14	0.267
Gender			0.825		0.714		0.658
Male	108	42		51		59	
Female	20	7		8		13	
Pathologic T-stage			0.654		**< 0.001**		**< 0.001**
Ta	92	39		22		25	
T1	14	3		4		26	
T2	21	7		19		18	
T3	0	0		14		3	
T4	1	0		0		0	
Readers 1			0.600		< 0.001		0.091
1	24	5		1		4	
2	57	26		25		39	
3	28	10		10		18	
4	9	5		5		3	
5	10	3		18		8	
Readers 2			0.680		< 0.001		0.070
1	24	5		2		5	
2	59	27		28		46	
3	26	9		6		11	
4	9	4		5		3	
5	10	4		18		7	
Degree of infiltration			0.954		**< 0.001**		0.058
NMIBC	104	40		26		50	
MIBC	24	9		33		22	

The data shown are the numbers of tumors

*p* < 0.05 is represented in bold

*MIBC* muscle-invasive bladder cancer, *NMIBC* non-muscle-invasive bladder cancer

### Performance of VI-RADS

For both Reader 1 and Reader 2, VI-RADS scores were standardized into binary categories: scores of 3, 4, or 5 were classified as muscle-invasive bladder cancer (MIBC), whereas scores of 1 or 2 were categorized as non-muscle-invasive bladder cancer (NMIBC) [30, 31].

Interobserver agreement was high, with Cohen’s kappa coefficients of 0.924, 0.877, and 0.863 for the internal validation cohort (Center 1) and the external validation cohorts (Centers 2 and 3), respectively.

As presented in Table 2, the AUC values for Reader 1 were as follows: 0.967 (95% CI: 0.871–0.997) for the internal validation cohort, 0.848 (95% CI: 0.731–0.929) for Center 2, and 0.901 (95% CI: 0.807–0.959) for Center 3. Reader 2 achieved AUCs of 0.971 (95% CI: 0.878–0.998), 0.885 (95% CI: 0.775–0.953), and 0.825 (95% CI: 0.718–0.905) in the corresponding validation cohorts. Representative biparametric MRI examples illustrating lesions across the full VI-RADS spectrum (scores 1–5), with corresponding T2WI, DWI, and ADC appearances, are shown in Fig. E1, together with the pathological labels and the MIBC probabilities predicted by the T2WI model and the Joint model for each lesion. For the three pathologically confirmed NMIBC lesions (VI-RADS scores 1–3), the Joint model assigned low probabilities, consistent with the pathological results, whereas the T2WI model assigned substantially higher probabilities, overestimating the likelihood of muscle invasion for the VI-RADS 2 and 3 lesions. For the two pathologically confirmed MIBC lesions (VI-RADS scores 4 and 5), both models assigned high probabilities.

**Table 2:** Prediction performance of the radiologist, deep learning models, and the joint model

Dataset	Model	AUC (SD)	ACC (SD)	SEN (SD)	SPE (SD)	*p*-value
Internal validation cohort (Center 1)	Reader 1	0.967 (-)	0.816 (-)	1.000 (-)	0.775 (-)	**< 0.001**
	Reader 2	0.971 (-)	0.837 (-)	1.000 (-)	0.800 (-)	**0.006**
	ResNet18	0.915 (0.017)	0.835 (0.034)	0.878 (0.035)	0.825 (0.044)	**< 0.001**
	Densenet121	0.926 (0.016)	0.843 (0.036)	0.856 (0.054)	0.840 (0.044)	**< 0.001**
	ResNet50	0.931 (0.020)	0.835 (0.056)	0.844 (0.057)	0.832 (0.078)	**< 0.001**
	Joint model	0.979 (0.007)	0.918 (0.033)	0.878 (0.035)	0.927 (0.045)	Reference
External validation cohort (Center 2)	Reader 1	0.848 (-)	0.762 (-)	0.788 (-)	0.731 (-)	**< 0.001**
	Reader 2	0.885 (-)	0.831 (-)	0.788 (-)	0.885 (-)	**< 0.001**
	ResNet18	0.830 (0.028)	0.758 (0.023)	0.715 (0.114)	0.812 (0.130)	**< 0.001**
	Densenet121	0.779 (0.028)	0.707 (0.032)	0.685 (0.183)	0.735 (0.179)	**< 0.001**
	ResNet50	0.856 (0.014)	0.792 (0.021)	0.755 (0.065)	0.838 (0.047)	**< 0.001**
	Joint model	0.907 (0.013)	0.842 (0.020)	0.821 (0.060)	0.869 (0.037)	
External validation cohort (Center 3)	Reader 1	0.901 (-)	0.825 (-)	0.867 (-)	0.809 (-)	**0.044**
	Reader 2	0.825 (-)	0.860 (-)	0.733 (-)	0.952 (-)	**< 0.001**
	ResNet18	0.834 (0.014)	0.749 (0.068)	0.800 (0.084)	0.733 (0.110)	**< 0.001**
	Densenet121	0.826 (0.011)	0.756 (0.037)	0.794 (0.097)	0.744 (0.072)	**< 0.001**
	ResNet50	0.841 (0.019)	0.732 (0.052)	0.835 (0.091)	0.700 (0.091)	**< 0.001**
	Joint model	0.916 (0.021)	0.842 (0.038)	0.859 (0.057)	0.836 (0.052)	

*p* < 0.05 is represented in bold

*AUC* area under the curve, *ACC* accuracy, *SEN* sensitivity, *SPE* specificity, *SD* standard deviation

### Performance of deep learning models

As shown in Table 2, over ten independent training runs, the Joint model achieved mean AUCs of 0.979 ± 0.007 for the internal validation cohort, 0.907 ± 0.013 for Center 2, and 0.916 ± 0.021 for Center 3. The T2WI model (ResNet-50) achieved mean AUCs of 0.931 ± 0.020, 0.856 ± 0.014, and 0.841 ± 0.019, respectively. The 3D ResNet-18 and 3D DenseNet-121 models showed lower mean AUCs in all cohorts (Table 2).

Paired *t*-test comparisons among the deep learning models revealed that the Joint model outperformed the T2WI model in all three cohorts, as well as the ResNet-18 and DenseNet-121 models. One-sample *t*-test comparisons showed that the Joint model significantly outperformed both Reader 1 and Reader 2 in all three validation cohorts.

**Fig. 3:**
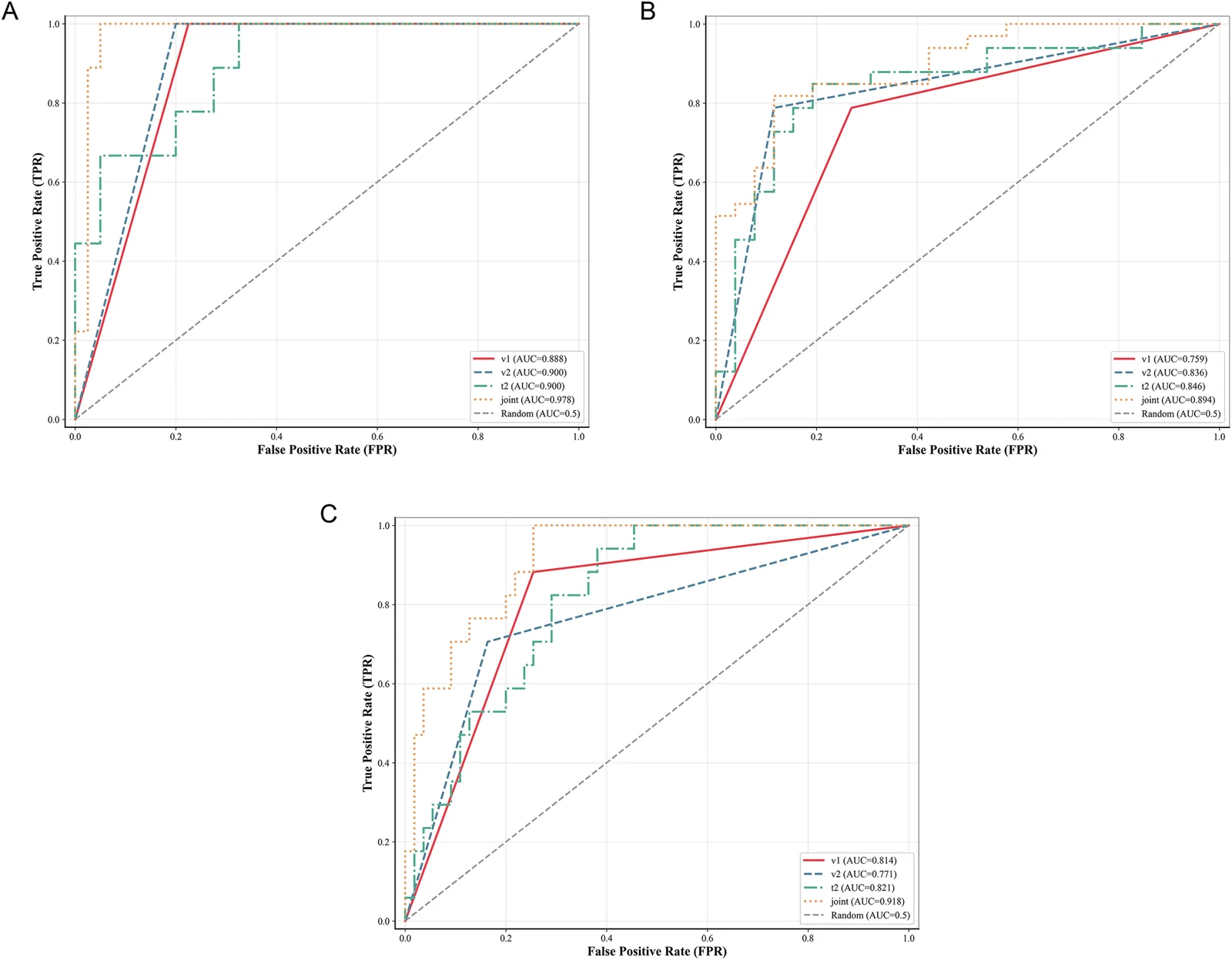
Receiver operating characteristic (ROC) curves for the T2WI model and Joint model and Reader 1 and Reader 2 in the internal validation cohort (Center 1) (**A**), external validation cohort (Center 2) (**B**), and external validation cohort (Center 3) (**C**)

**Fig. 4:**
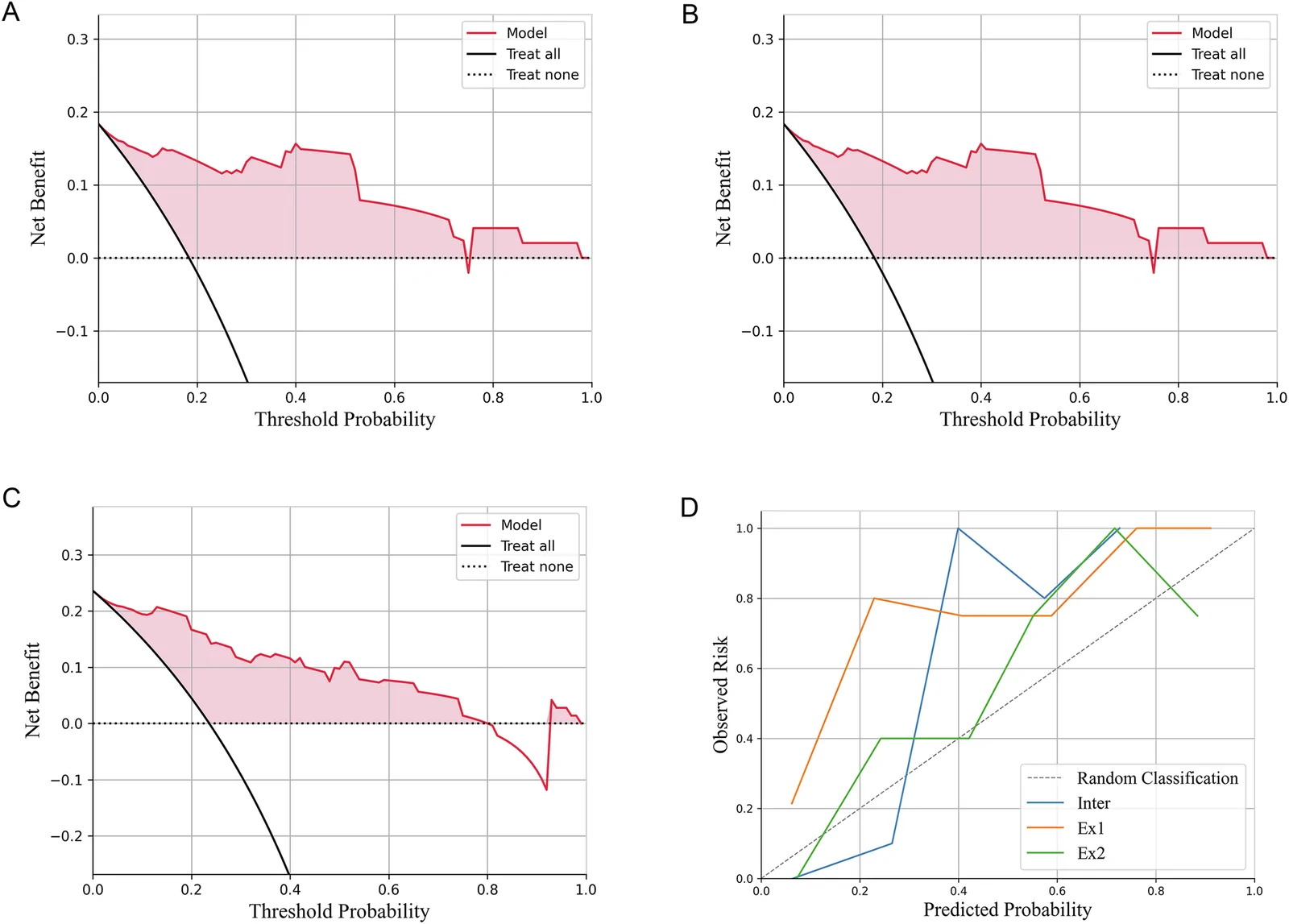
Decision curve analysis for the Joint model in the internal validation cohort (Center 1) (**A**), external validation cohort (Center 2) (**B**), and external validation cohort (Center 3) (**C**), and calibration curves of the Joint model for all validation datasets (**D**)

The ROC curves (Fig. 3), decision curve analysis (Fig. 4A–C), and calibration curves (Fig. 4D) were all derived from the selected model instance.

**Fig. 5:**
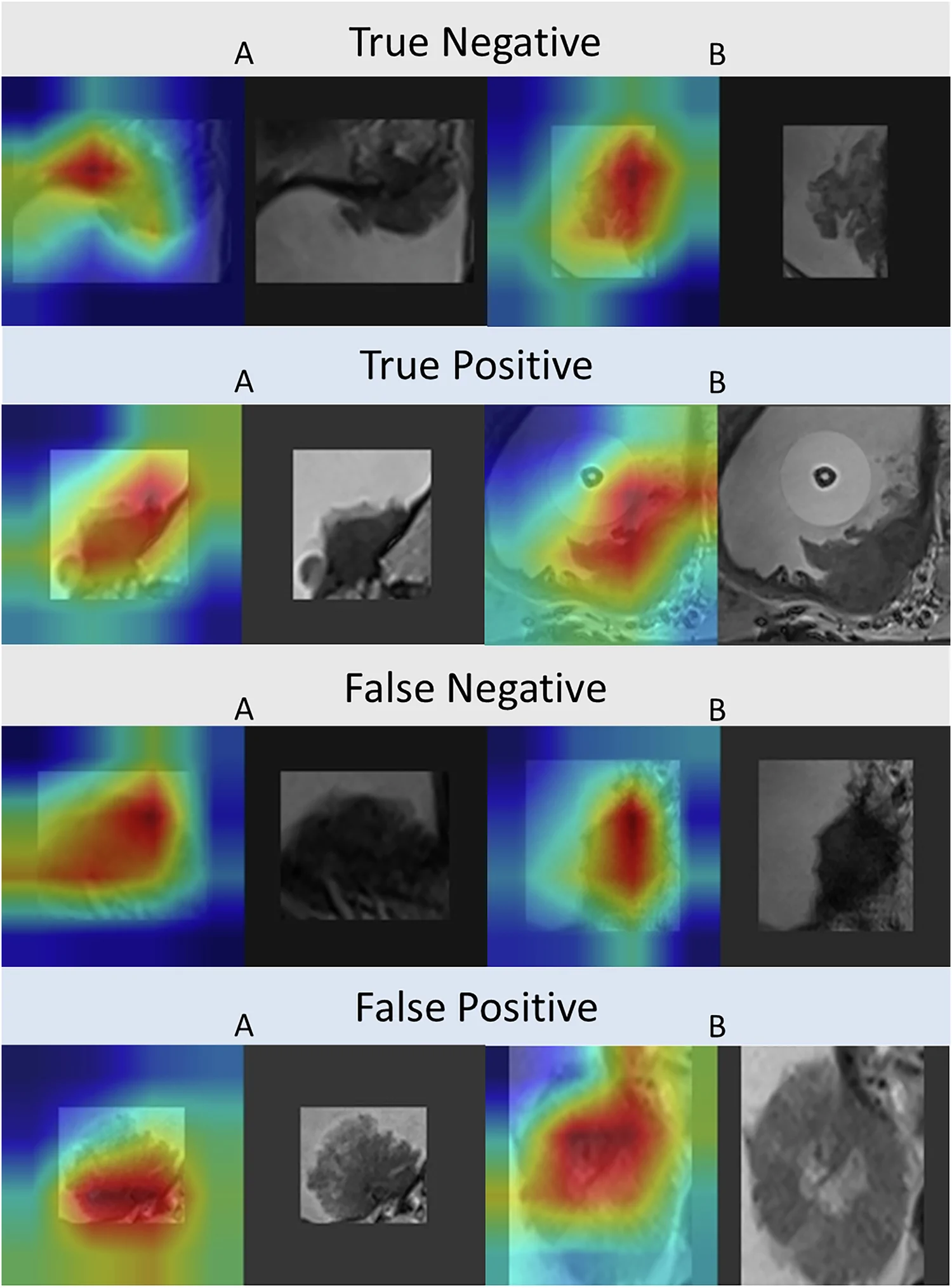
Grad-CAM maps calculated by ResNet. The red and yellow regions in the heatmaps represent areas with higher activation, whereas the blue and green regions represent areas with lower activation

**Fig. 6:**
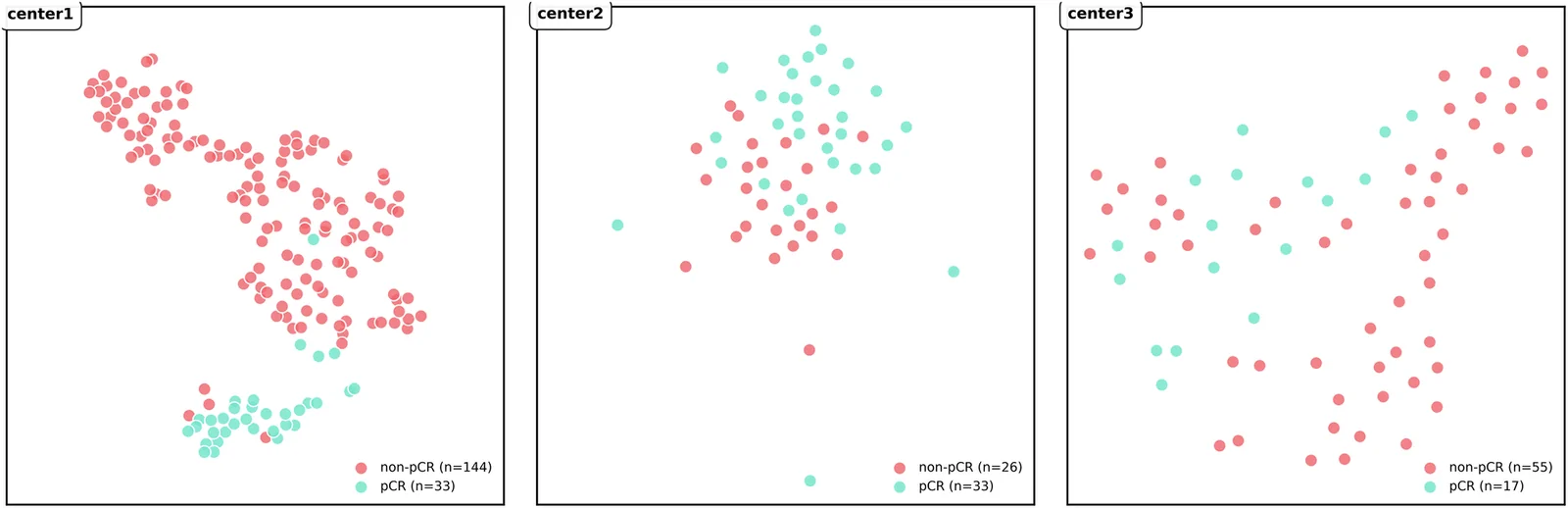
t-SNE dimensionality reduction plot, showing the feature distribution of the Joint model across different centers. The plot illustrates how the features are clustered and separated based on predicted outcomes

Decision curve analysis demonstrated that the Joint model provided substantial net clinical benefit across a range of threshold probabilities: 0–97% for the internal validation cohort, 0–96% for Center 2, and 0–76% for Center 3. The calibration curves demonstrated strong concordance between the predicted and observed probabilities in all three validation cohorts. Overlaid heatmaps on T2WI images (Fig. 5) revealed prominent activation in the tumor–bladder wall interface region, indicating that the deep learning model effectively captured features associated with tumor invasiveness. For true positive and true negative cases, the model’s attention heatmaps effectively covered tumor regions. For false-positive and false-negative cases, the heatmaps still localized to the tumor area, suggesting that prediction errors did not primarily arise from misplaced spatial attention but from misinterpretation of the attended morphological features. The t-SNE analysis showed that the features extracted by the Joint model distinguished MIBC from NMIBC (Fig. 6).

### Comparison of performance in tumors scoring VI-RADS 2 and 3

Using the same selected model instance, as shown in Table 3, for tumors assigned VI-RADS scores of 2 and 3 by Reader 1 in the internal validation cohort (Center 1), Reader 1 achieved an accuracy of 0.816. The T2WI model yielded an accuracy of 0.694, whereas the Joint model achieved an accuracy of 0.944. The Joint model demonstrated significantly higher accuracy compared to the T2WI model (*p* = 0.023), but not compared to Reader 1 (*p* = 0.109). The Joint model’s AUC (0.971, 95% CI: 0.852–0.999) was higher than that of the T2WI model (0.838, 95% CI: 0.678–0.939), although this difference was not statistically significant (*p* = 0.348). For tumors rated 2 or 3 by Reader 2 in the same cohort, Reader 2 had an accuracy of 0.837; the Joint model (0.944) significantly outperformed the T2WI model (0.694, *p* = 0.023) but did not differ significantly from Reader 2 (*p* = 0.180).

**Table 3:** Performance of the T2WI model and the Joint model in tumors scoring VI-RADS 2 and 3

Dataset	Model	AUC	ACC	SEN	SPE	*p*-value^a^	*p*-value^b^
Internal validation cohort (Center 1)	VI-RADS 2 and 3 in Reader 1						
	Reader 1	-	0.816	0.000	1.000	-	0.109
	T2WI model	0.838 (0.678, 0.939)	0.694	1.000	0.677	0.348	**0.023**
	Joint model	0.971 (0.852, 0.999)	0.944	1.000	0.941	-	-
	VI-RADS 2 and 3 in Reader 2						
	Reader 2	-	0.837	0.000	1.000	-	0.180
	T2WI model	0.838 (0.678, 0.939)	0.694	1.000	0.677	0.348	**0.023**
	Joint model	0.971 (0.852, 0.999)	0.944	1.000	0.941	-	-
External validation cohort (Center 2)	VI-RADS 2 and 3 in Reader 1						
	Reader 1	-	0.762	0.000	1.000	-	**0.003**
	T2WI model	0.773 (0.600, 0.897)	0.771	0.727	0.792	0.127	**0.031**
	Joint model	0.909 (0.763, 0.980)	0.942	0.818	1.000	-	-
	VI-RADS 2 and 3 in Reader 2						
	Reader 2	-	0.831	0.000	1.000	-	0.065
	T2WI model	0.733 (0.529, 0.865)	0.764	0.700	0.792	0.163	0.125
	Joint model	0.875 (0.716, 0.963)	0.912	0.700	1.000	-	-
External validation cohort (Center 3)	VI-RADS 2 and 3 in Reader 1						
	Reader 1	-	0.778	0.000	1.000	-	**0.021**
	T2WI model	0.778 (0.648, 0.877)	0.737	0.833	0.726	**0.049**	**0.049**
	Joint model	0.922 (0.819, 0.976)	0.895	1.000	0.882	-	-
	VI-RADS 2 and 3 in Reader 2						
	Reader 2	-	0.806	0.000	1.000	-	0.144
	T2WI model	0.788 (0.660, 0.885)	0.719	0.875	0.694	**0.028**	**0.021**
	Joint model	0.928 (0.828, 0.980)	0.895	1.000	0.878	-	-

*p* < 0.05 is presented in bold

*AUC* area under the curve, *ACC* accuracy, *SEN* sensitivity, *SPE* specificity

^a^ The significance level of the comparison of the AUC between the T2WI model and the Joint model according to Delong’s test

^b^ The significance level of the comparison of accuracy between the T2WI model and the Joint model according to McNemar’s test

In the external validation cohort from Center 2, for tumors rated 2 or 3 by Reader 1, Reader 1 had an accuracy of 0.762 and the T2WI model 0.771; the Joint model achieved an accuracy of 0.942. The Joint model demonstrated significantly higher accuracy than both Reader 1 (*p* = 0.003) and the T2WI model (*p* = 0.031). The Joint model’s AUC (0.909, 95% CI: 0.763–0.980) exceeded that of the T2WI model (0.773, 95% CI: 0.600–0.897), though the difference did not reach significance (*p* = 0.127). For tumors scored 2 or 3 by Reader 2 in Center 2, Reader 2 achieved an accuracy of 0.831 and the T2WI model 0.764; the Joint model achieved 0.912. The Joint model outperformed both Reader 2 and the T2WI model in accuracy, but neither comparison reached statistical significance (vs. Reader 2: *p* = 0.065; vs. T2WI model: *p* = 0.125). The Joint model’s AUC (0.875, 95% CI: 0.716–0.963) was higher than that of the T2WI model (0.733, 95% CI: 0.529–0.865), with no significant difference (*p* = 0.163).

In the Center 3 cohort, among tumors rated 2 and 3 by Reader 1, Reader 1 achieved an accuracy of 0.778, the T2WI model 0.737, and the Joint model 0.895. The Joint model achieved significantly higher accuracy than both Reader 1 (*p* = 0.021) and the T2WI model (*p* = 0.049). The Joint model also demonstrated a significantly higher AUC than the T2WI model (0.922, 95% CI: 0.819–0.976 vs. 0.778, 95% CI: 0.648–0.877; *p* = 0.049). For tumors scored 2 or 3 by Reader 2, Reader 2 had an accuracy of 0.806, the T2WI model 0.719, and the Joint model 0.895. The Joint model significantly outperformed the T2WI model in accuracy (*p* = 0.021) and AUC (0.928, 95% CI: 0.828–0.980 vs. 0.788, 95% CI: 0.660–0.885; *p* = 0.028). However, the accuracy difference between the Joint model and Reader 2 did not reach statistical significance (*p* = 0.144).

## Discussion

In this study, we developed a deep learning model using bp-MRI inputs (T2WI and DWI), referred to as the Joint model, for diagnosing muscle-invasive bladder cancer (MIBC). The Joint model integrates T2WI and DWI through a low-rank Hadamard bilinear fusion module with deep supervision, and was compared with radiologists’ VI-RADS assessments and three single-sequence deep learning models trained solely on T2WI. Our findings demonstrate that the Joint model outperformed both the VI-RADS scoring and the T2WI-based model in predicting muscle invasion in patients with bladder cancer. Notably, for lesions scored as VI-RADS 2 or 3, the Joint model significantly outperformed the T2WI model in diagnostic accuracy and showed strong generalizability across external validation cohorts.

Our results demonstrated strong interreader agreement of VI-RADS assessments: the Cohen’s kappa coefficient between Reader 1 and Reader 2 was 0.924 in the internal validation cohort from Center 1 and 0.877 and 0.863 in the external validation cohorts from Centers 2 and 3, respectively—levels consistent with previous studies [32, 33]. In terms of diagnostic performance, the Joint model achieved mean AUCs of 0.979 ± 0.007 in Center 1’s internal validation cohort, 0.907 ± 0.013 in the external validation cohort from Center 2, and 0.916 ± 0.021 in Center 3. These results numerically exceeded those of Reader 1 (0.967, 0.848, and 0.901) and Reader 2 (0.971, 0.885, and 0.825) in all cohorts. Statistically, the Joint model significantly outperformed both readers in the internal validation cohort (Reader 1: *p* < 0.001; Reader 2: *p* = 0.006) and in Center 2 (both *p* < 0.001). In Center 3, the Joint model significantly outperformed Reader 2 (*p* < 0.001) and Reader 1 (*p* = 0.044). Furthermore, the Joint model significantly outperformed all three single-sequence T2WI-based models in all cohorts (paired *t*-test, all *p* < 0.001). The fact that all single-sequence models—regardless of backbone complexity—lagged significantly behind the Joint model suggests that T2WI alone is insufficient for robust cross-institutional prediction, and that the integration of DWI through the proposed low-rank fusion module contributes incremental diagnostic value.

In studies where only the T2WI sequence was used, Li et al [15] achieved an AUC of 0.932 in both internal and external validations via a multitask learning approach, although their method relied on labor-intensive manual annotation of tumor regions. Multisequence MRI modeling often outperforms single-sequence approaches, as different sequences provide complementary lesion information. For example, T2WI effectively presents the anatomical structure and morphological features of bladder lesions, whereas DWI provides functional insights by quantifying the diffusion characteristics of water molecules in tissues [10]. As illustrated in Fig. E1, tumor lesions appeared visually brighter and more conspicuous on DWI than on T2WI. For the three NMIBC lesions (VI-RADS 1–3), the Joint model’s probabilities remained low, whereas the T2WI model overestimated the likelihood of muscle invasion, most notably for the VI-RADS 2 (0.6908 vs. 0.3837) and VI-RADS 3 (0.7576 vs. 0.3824) lesions. Of particular interest is the VI-RADS 3 lesion: although the binary VI-RADS categorization (scores 3–5 as MIBC) would classify it as muscle-invasive, the Joint model’s low probability was consistent with the pathological diagnosis of NMIBC. These examples suggest that integrating DWI functional information helps the model avoid overcalling morphologically ambiguous lesions that are prone to interobserver inconsistency. Yu et al [16] reported high predictive performance using an mp-MRI-based deep learning model (AUC = 0.928), and their bp-MRI-based model (AUC = 0.927) demonstrated similarly high performance. Another study [32] further confirmed that bp-MRI offers diagnostic accuracy comparable to mp-MRI for assessing muscle invasion. In contrast, our Joint model achieved strong diagnostic performance while avoiding gadolinium-based contrast agent risks [9] and reducing annotation complexity. This was enabled by the low-rank Hadamard bilinear fusion module, which was selected for its ability to explicitly model pairwise second-order interactions between T2WI (anatomical) and DWI (functional) features, directly capturing their complementary cross-sequence relationship while controlling model complexity through low-rank factorization [24, 25]. A deep supervision strategy further ensures that each encoder branch learns discriminative unimodal representations before fusion, preventing over-reliance on a single modality. Collectively, these design choices allow the model to exploit DWI complementary information without excessive parameters, facilitating clinical deployment in resource-limited settings.

We performed a subgroup analysis comparing the performance of the Joint model and the T2WI model in tumors with VI-RADS scores of 2 and 3. In the internal validation cohort, the Joint model achieved significantly higher accuracy than the T2WI model (0.944 vs. 0.694, McNemar’s test *p* = 0.023) and numerically outperformed both readers. Across external cohorts, the Joint model consistently yielded higher AUCs than the T2WI model in all reader-defined VI-RADS 2–3 subgroups, with statistically significant differences observed in Center 3 (DeLong’s test, *p* = 0.049 and *p* = 0.028). Notably, in Center 2, the Joint model significantly outperformed Reader 1 (*p* = 0.003) and the T2WI model (*p* = 0.031) in the Reader 1–defined subgroup. These findings suggest that the integration of DWI functional information through the proposed fusion architecture provides particular value for morphologically ambiguous lesions that are prone to interobserver inconsistency. The Joint model achieved comparable overall AUCs across the two external cohorts (0.907 in Center 2 vs. 0.916 in Center 3), with only slight performance decreases in the VI-RADS 2–3 subgroup at Center 2. This minor variation indicates that the model maintains acceptable cross-vendor generalizability.

Grad-CAM analysis of false-negative and false-positive cases showed that heatmaps remained focused on the tumor region (Fig. 5), indicating that errors arose from misinterpretation of attended features rather than from spatial attention failure. In false-negative case B (Center 1), focal muscle invasion was accompanied by abundant peritumoral inflammatory hyperplasia, and the inflammatory tissue was nearly isointense to detrusor muscle on T2WI. This caused the model to misread the tissue plane as an intact muscle barrier despite correct heatmap localization. In false-positive case A (Center 1), a large hypervascular tumor with increased peritumoral small vessels produced T2WI heterogeneity that enlarged the apparent tumor boundary, and the model misinterpreted this expanded tumor-muscle contact as transmural invasion. These cases highlight a key limitation of Grad-CAM: it confirms anatomical attention but cannot reveal why correctly localized lesions are misclassified [34].

Our study had several limitations. First, the retrospective design inherently introduces selection bias and limits control over imaging protocols, data quality, and standardization of collection procedures across centers. A prospective multicenter study with predefined inclusion criteria and standardized acquisition protocols is warranted to further validate the model’s clinical utility and generalizability. Second, manual delineation of volumes of interest (VOIs) on both sequences was necessary for model training. The development of a fully automated framework for MIBC diagnosis is a promising direction for future work. Third, the current feature fusion strategy is relatively rudimentary, and only two imaging sequences are incorporated. Incorporating DCE for a direct comparison with mp-MRI and exploring more advanced fusion methods are important future directions. Additionally, Grad-CAM only localizes model attention, and more interpretable methods are needed to explain the actual decision logic.

## Conclusion

In conclusion, we propose the Joint model that integrates T2WI and DWI sequences for the diagnosis of MIBC. This model outperformed both VI-RADS and the T2WI model in multicenter validation, and showed improved performance for lesions scored VI-RADS 2 and 3 compared with the T2WI model and VI-RADS assessment. These strengths highlight the clinical value of the Joint model for the reliable and noninvasive evaluation of muscle invasion in patients with bladder cancer.

## Supplementary information

ELECTRONIC SUPPLEMENTARY MATERIAL

## Data Availability

The data underlying this article will be shared on reasonable request to the corresponding author.

## Abbreviations

ACC: Accuracy
AUC: Area under the receiver operating characteristic curve
bp-MRI: Biparametric magnetic resonance imaging
CI: Confidence interval
DCE: Dynamic contrast-enhanced imaging
DL: Deep learning
DWI: Diffusion-weighted imaging
EFL: Equalized focal loss
Grad-CAM: Gradient-weighted class activation mapping
MIBC: Muscle-invasive bladder cancer
mp-MRI: Multiparametric magnetic resonance imaging
NMIBC: Non-muscle-invasive bladder cancer
SD: Standard deviation
SEN: Sensitivity
SPE: Specificity
t-SNE: t-distributed stochastic neighbor embedding
T2WI: T2-weighted imaging
TURBT: Transurethral resection of bladder tumor
VI-RADS: Vesical Imaging-Reporting and Data System
VOI: Volume of interest

## Declarations

### Study design

- Retrospective
- Multicenter
- Observational
