# Supplementary material for: Biparametric MRI with deep learning for the preoperative prediction of muscle invasion in patients with bladder cancer: a multicenter study
Source: Insights Imaging. 2026 Nov 9;17:289. doi: 10.1186/s13244-026-02430-1 (PMC13748144; doi:10.1186/s13244-026-02430-1)
Supplement: Supplementary file 1 [file 13244_2026_2430_MOESM1_ESM.pdf]

Biparametric MRI with deep learning for the preoperative prediction of muscle invasion in patients with bladder cancer: a multicenter study

ELECTRONIC SUPPLEMENTARY MATERIAL

A1. Image Acquisition

All patients underwent multiparametric magnetic resonance imaging (mpMRI) using a 3.0 Tesla MRI scanner. Prior to the examination, patients were instructed to fast for 4–6 hours. Image acquisition was performed with patients in the supine position and a bladder filling volume maintained between 250 and 300 mL. The MRI sequences, encompassing T2-weighted imaging (T2WI), diffusion-weighted imaging (DWI), and dynamic contrast-enhanced MRI (DCE-MRI), were all acquired in the axial plane.

For DCE-MRI, gadopentetate dimeglumine (Gd-DTPA) was administered as the contrast agent at a dosage of 0.2 mmol/kg, with an injection rate of 2 mL/s. Image acquisition included one pre-contrast phase and nine post-contrast dynamic phases, with post-contrast scanning commencing 15 seconds after contrast administration. The specific MRI acquisition parameters for the two centers are detailed in Supplementary Table E1.

A2. Image Preprocessing

All VOIs were manually delineated by a radiologist and verified for complete tumor coverage. To assess inter-observer reliability, a second radiologist with experience in genitourinary imaging independently annotated a randomly selected subset; the intraclass correlation coefficient (ICC, two-way random model, absolute agreement) yielded a mean ICC of 0.964. Given the computational constraints of deep learning (DL), 3D volumes of interest (VOIs) were processed as image patches rather than full volumes. Each VOI was partitioned into patches of size 128 × 128 × 8. A sliding window approach was adopted along the z-axis with a stride of 5 slices.

The patch extraction strategy was as follows: If a VOI contained fewer than 8 slices along the z-axis, zero-padding was applied to create a single valid patch. For VOIs exceeding 8 slices, patches were extracted sequentially with the defined stride. To ensure full coverage of the tumor volume, the final patch was anchored to the last slice of the VOI (i.e., capturing the last 8 slices), regardless of overlap with the preceding patch. Crucially, the same patch extraction logic was applied to both T2WI and DWI sequences to ensure spatial correspondence.

For inference aggregation, when multiple patches were generated from a single lesion, the model first predicted the probability for each patch. These probabilities were then averaged to yield the final malignancy risk for the patient.

To facilitate model convergence, image intensity values were normalized to the range [0, 1]. Data augmentation was applied to all training patches to strictly mitigate overfitting and enhance generalizability. Techniques included horizontal and vertical flipping, cropping, affine transformations (scaling and translation), image sharpening, contrast adjustment, and Gaussian noise injection.

A3. Statistical Analysis

Continuous variables were evaluated for normality using the Kolmogorov–Smirnov test or Shapiro–Wilk test. Normally distributed variables were expressed as mean ± standard deviation (SD) and compared using the independent samples t-test. Categorical variables were presented as frequencies and percentages and compared using the chi-square test. Model calibration was assessed using the Hosmer–Lemeshow goodness-of-fit test. Decision curve analysis (DCA) was conducted to evaluate the clinical utility of the diagnostic models by quantifying the net benefit at different threshold probabilities. A two-sided p-value of < 0.05 was considered statistically significant. (Note: Additional comparisons of AUC and accuracy using DeLong's test and McNemar's test are described in the main text methods.)

Table E1. MRI scanning protocols

Parameters	Center 1			Center 2			Center 3		
MR scanner	MAGNETOM Skyra, Siemens, Germany			UMR 780, United Imaging Healthcare, Shanghai, China			MAGNETOM Skyra, Siemens, Germany		
sequence	T2WI	DWI	DCE-MRI	T2WI	DWI	DCE-MRI	T2WI	DWI	DCE-MRI
TR (ms)	7500	3400	3.31	4000	4500	4.60	3200	5400	4.03
TE (ms)	101	61	1.22	120	61	1.41	120	82	1.29
Flip angle (degree)	90	90	13	90	90	10	90	90	10
FOV (cm)	20	20	30	20	36	30	20	20	30
Matrix	320*320	102*170	192*192	336*269	128*128	256*256	256*256	132*132	320*240
Slice thickness (mm)	4	4	2	3	5	2	3.5	4	1
Slice gap (mm)	0.4	0	0	0.6	1	-1	0.7	0.8	0.2
Number of excitations	2	2,8	1	1.5	4,16	1.5	2	2,8	1
B values	0，1000s/mm^2			0，1000s/mm^2			0，1000s/mm^2		

TR = repetition time; TE = echo time; T2WI = T2-weight imaging; DWI = diffusion-weighted imaging; DCE-MRI = dynamic contrast-enhanced MRI; FOV = field of view.

Table E2. Patient data characteristics (Patient-Based Summary).

Characteristics	Number of patients				
	Center 1 (138)	Center 2 (57)	p value	Center 3 (59)	p value
**Age (years)**	65.01±11.41	64.00±12.09	0.582	64.22±9.79	0.649
**Sex**			0.648		0.961
Male	115	49		49	
Female	23	8		10	
**Patient's tumor number**			0.047		0.680
Single	120	55		50	
Multiple	18	2		9	
**Type of surgery**			**<0.001**		0.002
TURBT	129	38		46	
RC	9	19		13	

The data shown are the numbers of patients. Abbreviations: TURBT, Transurethral Resection of Bladder Tumor; RC, Radical Cystectomy.

$p < 0.05$ is represented in bold.

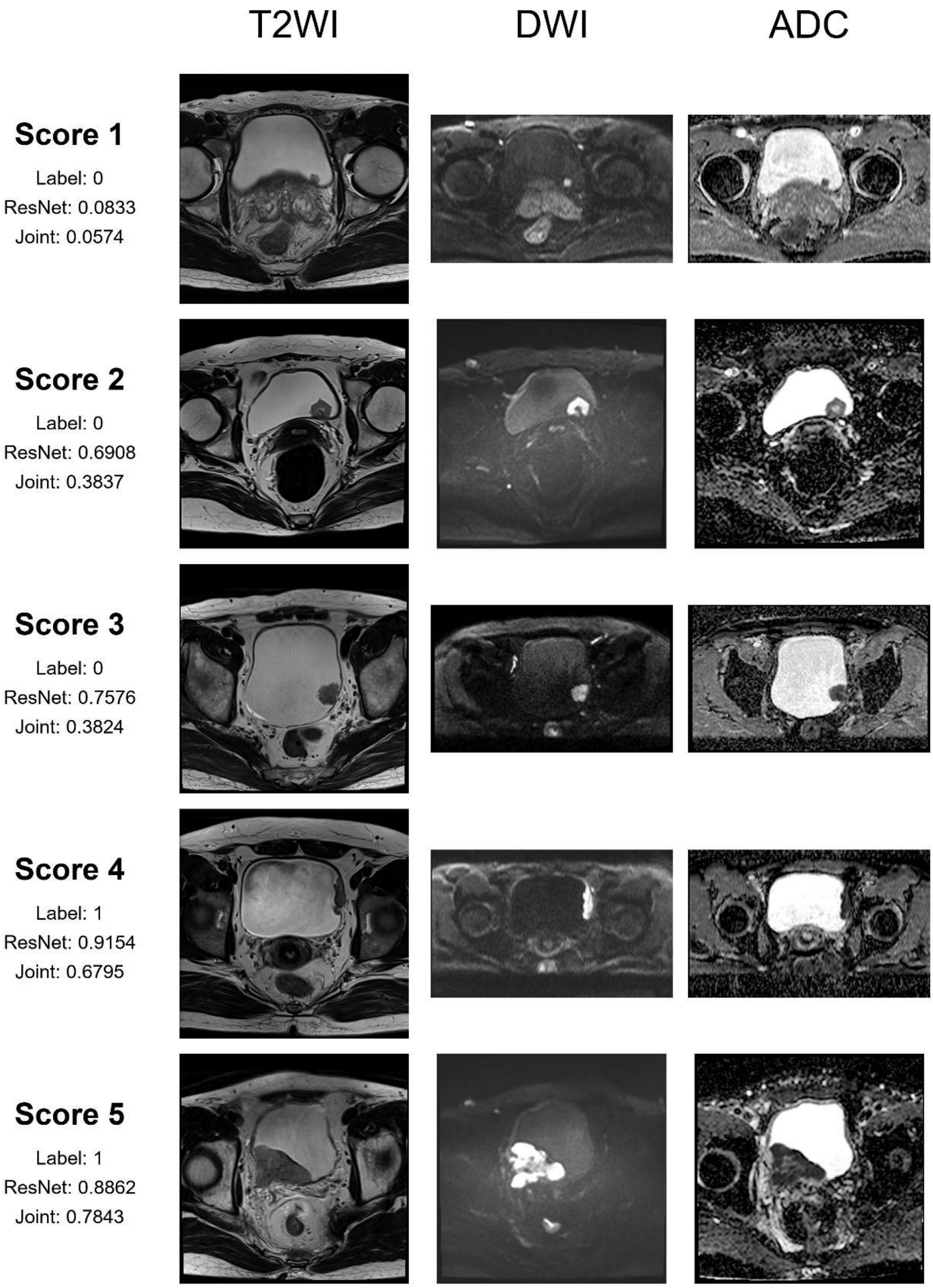

Figure E1. Representative biparametric MRI examples of bladder cancer across the VI-RADS spectrum. Each row shows a lesion assigned a VI-RADS score from 1 to 5, with corresponding T2-weighted imaging (T2WI), diffusion-weighted imaging (DWI), and apparent diffusion coefficient (ADC) map.
